# Evaluation of Crossover Sign in Pelvis Models Made with a Three-Dimensional Printer

**DOI:** 10.1155/2022/4665342

**Published:** 2022-05-26

**Authors:** Amirhossein Salimi, Seyed Peyman Mirghaderi, Mohammad Javad Gholamzadeh, Reihane Qahremani, Alireza Hadizadeh, Reza Shahriarirad, Hesan Jelodari Mamaghani, Javad Dehghani, Maryam Salimi

**Affiliations:** ^1^Student Research Committee, Shahid Sadoughi University of Medical Sciences, Yazd, Iran; ^2^Students' Scientific Research Center (SSRC), Tehran University of Medical Sciences, Tehran, Iran; ^3^Student Research Committee, Shiraz University of Medical Sciences, Shiraz, Iran; ^4^School of Medicine, Tehran University of Medical Sciences, Tehran, Iran; ^5^Research Center for Advanced Technologies, Cardiovascular Medicine, Cardiovascular Diseases Research Center Institute, Tehran University of Medical Sciences, Tehran, Iran; ^6^Bone and Joint Diseases Research Center, Department of Orthopaedic Surgery, Shiraz University of Medical Sciences, Shiraz, Iran

## Abstract

**Introduction:**

Investigation of the crossover sign (COS) in different degrees of tilt in pelvises made by three-dimensional printing of CT scans among patients with normal hip versions was carried out.

**Methods:**

Radiology CT scans of 8 normal pelvises reconstructed in 3D and the effect of sequential tilting on the presence of the false-positive COS in 48 radiographs were investigated.

**Results:**

The COS was seen in 77% of the AP radiographs during tilt changes. The average distance between the tip of the coccyx and the symphysis pubis was 32.06 ± 10.99 mm. Also, COSs were present in all radiographs from 6 degrees tilt and above.

**Conclusion:**

Minor tilting of the pelvis can result in a false-positive crossover sign on AP plain radiographs.

## 1. Introduction

The spatial orientation of the acetabulum, including its version and inclination, is an important anatomical feature of the hip joint [1]. Some of the major complications of hip arthroplasty include hip dislocation, impingement, and polyethylene abrasion and are related to improper position of acetabular hip prosthesis [2, 3]. In addition to the clinical significance of the acetabular position in joint replacement surgeries, acetabular version abnormalities can lead to hip pain and disability. Likewise, one of the most important causes of hip pain in young adults is the impingement between the head of the femur and the acetabulum. Furthermore, the retroversion of the acetabulum, which can be present globally or localized, can significantly increase the mentioned impingement [4, 5].

Normally, the anterior wall of the acetabulum is placed medially with regards to the posterior wall of the acetabulum, and the two walls meet at the top of the acetabulum [6, 7]. In a plain AP radiograph, if the anterior rim of the acetabulum intersects the posterior rim below the upper margin of the acetabulum, it is called a crossover sign (COS). Evaluation of femoral head coverage and acetabular orientation contributes to preoperative planning and decision-making of reorientation procedures [8]. Posterior and anterior femoral head coverage on an anteroposterior (AP) hip radiograph can be assessed by tracing the posterior and anterior rim contours. The increased risk for impingement followed by a retroverted acetabulum with excessive anterior coverage can be diagnosed in radiographs by the COS. Although the COS is the well-known radiographic sign for acetabular retroversion, there are controversial opinions about this sign's clinical compliance and accuracy in the diagnosis of global or local retroversion [9, 10].

In this study, we aimed at investigating the crossover sign in different degrees of tilt in pelvises made by three-dimensional printing of CT scans among patients with normal hip versions.

## 2. Materials and Methods

### 2.1. Patient Selection

**Figure 1:**
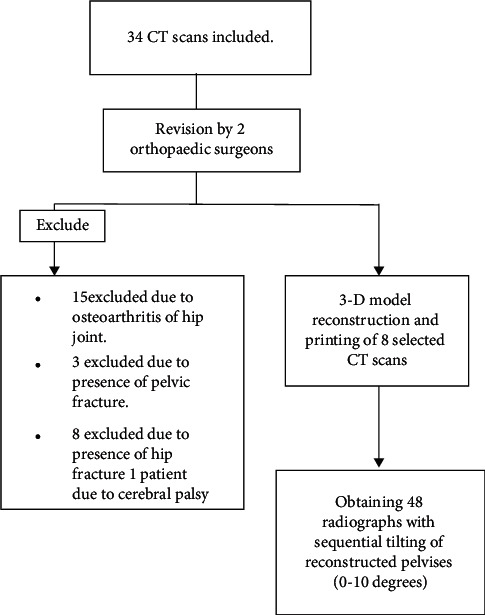
Flowchart of the included CT scans of the study.

To obtain normal pelvic radiography, two expert orthopaedic surgeons reviewed radiology CT scans from the picture archiving and communication system (PACS) database from 2013 to 2015. The inclusion criteria were patients above 20 years of age, with normal pelvic CT scans in terms of inclination and anteversion of the acetabulum up to a limit of one standard deviation from the average proportion (calculated as 12.5 ± 4.2 degrees for acetabular anteversion and below 8.3 degrees for true global retroversion). Exclusion criteria were technically inadequate imaging (CT scan images with slice thickness greater than 0.6 mm and images which were acquired within maximum voltage and current strength) or any signs of abrasion, osteoarthritis of the hip joint, or the presence of any fractures in the pelvis, hip, or sacrum bones. Of all 34 CT scans primarily included in this study, 15 were excluded due to osteoarthritis of the hip joint, 3 were excluded due to the presence of pelvic fracture, and 8 CT scans were excluded due to the presence of hip fracture (Figure 1). This study was approved by the Ethics Committee of Shiraz University of Medical Sciences. All patients' information was anonymized, and only the patients' age was used among the inclusion criteria. No prior study allowed power calculation. Therefore, the final sample size was chosen to obtain the pilot data. Eventually, a total of 8 pelvic CT scans were selected for this preliminary communication.

### 2.2. Three-Dimensional Print Model Generation

All the materials used in this research were purchased. No further purification has been applied to them. Biograde thermoplastic polyurethane (TPU), polylactic acid (PLA), and polyvinyl alcohol (PVA) were purchased from Wenzhou City Sanho Co., Ltd., China. Dichloromethane, dimethylformamide, and double-distilled water were used as the solvent for PLA, TPU, and PVA, respectively.

The DICOM images from the hip CT studies (axial soft tissue algorithm with 0.6 mm slice thickness) for each patient were extracted from ININITT PACS software (INFNITT Healthcare Co., South Korea). The maximum voltage and current strengths were 120 kV and 30 mA, respectively. The images were uploaded to MIMICS 19 software, reconstructed in 3D, and saved in the stereolithography (STL) format. Each 3D file was then printed to a 1 : 1 scale with the help of a bioprinter (Ultimaker S5) with an accuracy of 300 microns and a low-density PLA filament. The bioprinter was set up according to the following protocol: average nozzle temperature about 230°C, bed heat 70°C, and the height layer 0.05 mm with a print speed of 30 mm/s. We created the model using the following good manufacturing practice (GMP) protocols.

### 2.3. Study Design

**Figure 2:**
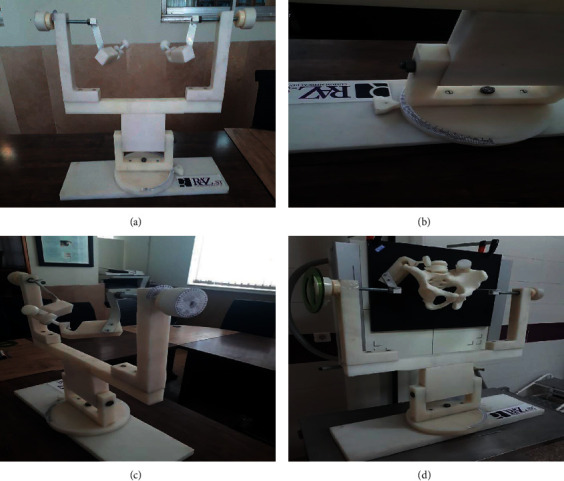
(a) Pelvic holder. (b) The degree of rotation maintenance. (c) Tilt degree maintenance. (d) Utilizing the pelvic holder for roentgenography of the pelvic model.

After preparing eight three-dimensional models, X-ray imaging of the models was performed according to the standard methods [11]. Each pelvic model made by the 3D printer was placed on a premade holder (Figure 2(a)). This device consists of two holders connected to the wing of the ileum of the pelvis by a clamp that fixes the model in place. This device also has a calibrated rotating base that allows us to create the desired internal and external rotation (Figure 2(b)). There are also two calibrated handles on the device's top that allow anterior and posterior tilts (Figure 2(c)).

Zero rotation and tilt determine when the pelvis is positioned parallel to the coronal axis of the body, and the anterior superior iliac spine on both sides is aligned with the pubic symphysis. In the initial radiograph, which is considered the baseline of rotation and tilt, the tip of the coccyx should be parallel to the pubic symphysis and should be one to three centimeters apart. Also, the obturator foramina on both sides must be perfectly symmetrical.

After determining the baseline point of rotation and tilt in the pelvis (assigned as zero points), the effect of pelvic tilt in the normal range of standard pelvic imaging resulting in a one to three centimeters gap from the coccyx to the pubic symphysis is measured on the presence or absence of the crossover sign in the acetabulum on both the sides. The effect of pelvic deviation to the sides in a range that does not lead to obvious asymmetry in the obturator cavity and other pelvic landmarks is also measured based on the crossover sign. These changes should not be to an extent to violate the normal pelvic radiography criteria and should be classified as an abnormal radiograph by the orthopaedist [11].

To investigate the effect of pelvic tilt on the occurrence of a positive COS, each of the 8 pelvises made by a 3D printer was taken in 6 standard graphics conditions. Since the application of posterior tilt reduced the distance between the coccyx and symphysis pubis to less than 1 cm and violated standard imaging, only changes in anterior tilt were evaluated. Tilt changes were applied in the range of 2–10 degrees anteriorly by increasing tilt by 2 degrees in each radiography. Hence, a total of 48 images were taken from the eight pelvic models. In all these radiographs, rotation was not applied and preserved at zero degrees, while our variable was considered anterior tilt.

All descriptive statistics were reported using IBM SPSS (version 19).

### 2.4. Ethical Approval

This experimental HIPAA-compliant study used retrospective data and was approved by the institutional review board, which waived the requirement for informed consent.

## 3. Results

The mean age of cases was 34.8 ± 8.5 years (minimum 25 and maximum 50 years old) (Table 1). The distance between the coccygeal tip and the symphysis pubis (C-S distance) was evaluated based on gender; the average distance in male pelvic models was 32.83 ± 12.59 mm (range 10–57), while in female models, it was 31.29 ± 9.33 mm (range 12–48). The average anteversion of reconstructed CT scans of included cases was 11.7 ± 1.1 degrees.

**Figure 3:**
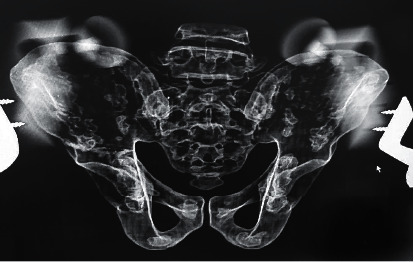
Pelvis with zero rotation and tilt with no positive crossover sign.

**Figure 4:**
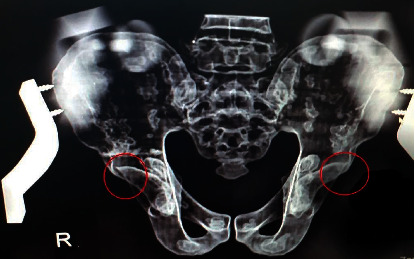
The same pelvis with a change in tilt (4 degrees) and the appearance of a positive crossover sign.

**Figure 5:**
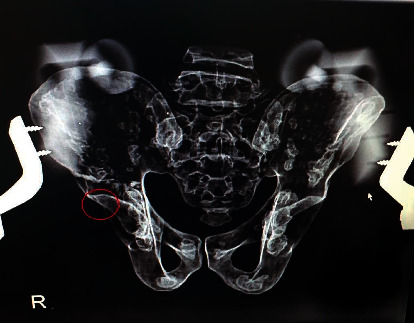
Appearance of the crossover sign in the same pelvic model after applying minimal rotation.

Of all 48 images, a crossover sign was seen in 77% of the radiographs (Table 1). As to the effect of the rotation change on the appearance of the COS, it was observed that the crossover sign appeared in all pelvic models with a slight change in rotation (2 degrees). Figures 3, 4, and 5 show the close-up view of radiographs of one of the pelvises, which did not have a crossover sign in zero-degree tilt (Figure 3) but was present after applying posterior tilt (Figure 4) or slight rotation (Figure 5).

**Figure 6:**
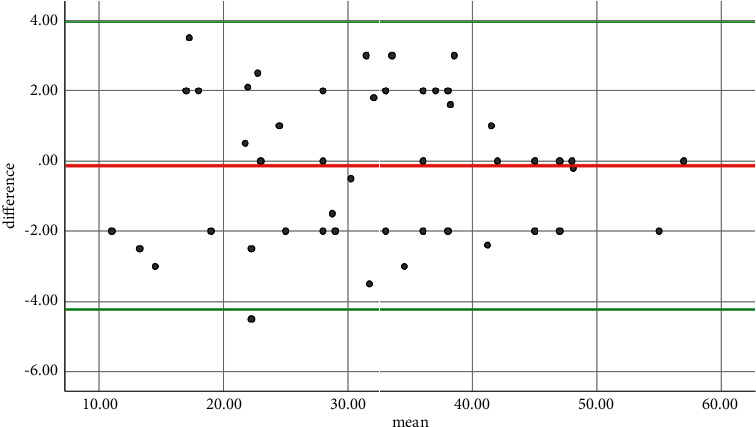
The Bland–Altman plot showing no significant difference in measurements.

The distance between the tip of the coccyx and the symphysis pubis was calculated separately with the changes in tilt in the radiographs taken from each pelvic model by two orthopaedic surgeons blinded to the study, the Bland–Altman agreement between these two measurements (Figure 6). The mean and standard deviation of this distance in the total radiographs taken in our study were 32.06 and 10.99 mm, respectively (Table 1).

**Figure 7:**
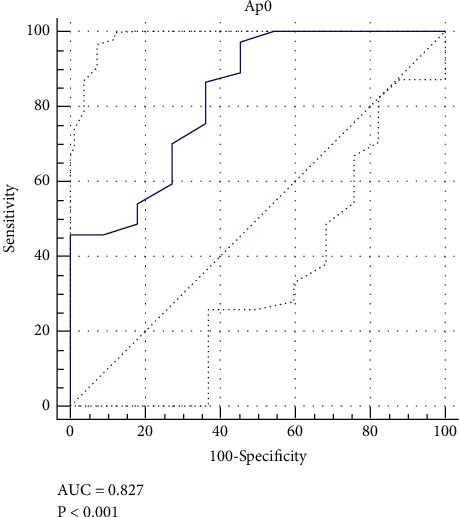
The receiver operating characteristic (ROC) curve displaying the cutoff point of 19 mm for C-S distance.

As given in Table 1, 37 (77%) radiographs had a positive COS. Radiographs that included the positive COS along with a normal coccygeal tip to the symphysis pubis distance (10–30 mm) were distinguished (Table 1), which comprised 14 (29.2% of total and 38% of radiographs with the positive COS) images. Therefore, 62% of the false-positive crossover signs can be excluded by measuring the C-S distance. The receiver operating characteristic (ROC) curve (Figure 7) was drawn to find an acceptable range of C-S distance with the lowest probability of a false-positive crossover sign. The cutoff point of 19 mm was reported for C-S distance. Out of the 8 pelvic models, 6 (75%) had at least one true pelvic radiography with a positive crossover sign in the presence of normal C-S distance. According to the crossover sign, the sensitivity and specificity of plain radiographs were in normal C-S distance at different tilt angles in the diagnosis of retroversion (Table 2). Furthermore, the receiver operating characteristic (ROC) curve displays the cutoff point of 19 mm for C-S distance (Figure 7).

**Figure 8:**
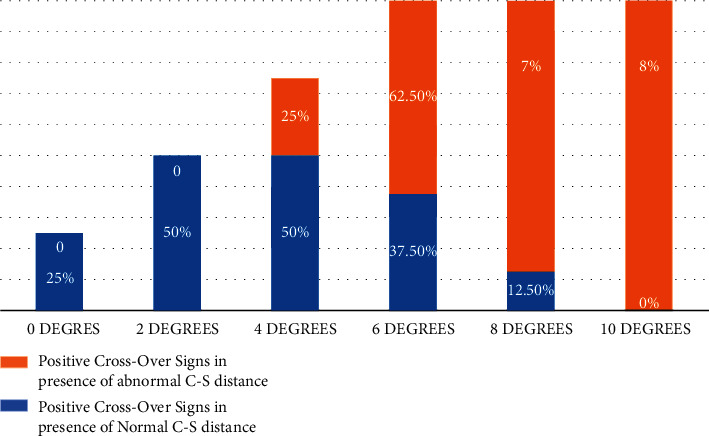
The presence of the crossover sign in pelvic tilt based on normal and abnormal C-S distance.

At 0 degrees (native position), both COSs were at normal C-S distance; while at 10-degree tilt, all radiographs had a positive COS in the presence of abnormal C-S distance. Also, crossover signs were present in all radiographs from 6 degrees tilt and above (Figure 8).

## 4. Discussion

We found that the COS was falsely positive in 77% of radiographs which extended from 25% in the native position to 100% at 6° and above. In other words, the false-positive COS even in the normal acetabulum at greater than and equal to 6° is inevitable. Tannast et al. reported that the presence of the errant COS increased to 100% by tilting the pelvis by 9° [12]. Ross et al. also indicated the rise in the mentioned percentage from 48% in the native position to 86% at a tilt of 10° [13]. Similar evidence was disseminated repeatedly by other authors regarding the increase in the false-positive COS by rotating or tilting the pelvis. In other words, the sensitivity and specificity of the COS for the evaluation of acetabular retroversion will change significantly depending on the deviation from the normal position in hip radiography [14–17]. In contrast, some studies gave great diagnostic validity to this sign [18, 19]. For instance, Jamali et al. reported the sensitivity and specificity of approximately 90% for the COS [18]. Based on the points mentioned above, it would seem that the COS, due to high dependency on the standard radiography position, cannot solely be used to evaluate acetabular retroversion. Furthermore, the simultaneous use of other radiologic markers, including the ischial spine sign and posterior wall sign, will contribute to accurate diagnosis of acetabular retroversion.

The vertical distance of the coccyx to pubic symphysis was introduced as a parameter to differentiate standard radiographs [14]. Distances outside the normal range (10–30 mm) indicate pelvic tilt and nonstandard radiographs. The cumulative evidence at present displays that the false-positive COS can be distinguished in almost 62% by measuring the mentioned distance. Moreover, the forgoing distance was similar between males and females; thus, the results can be applied to both genders. Other studies also have the same point of view on the value of measuring the distance of the coccyx to pubic symphysis in denoting a neutrally rotated pelvis and evaluation of standard radiographs [20, 21]. On the other hand, around 38% of false-positive radiographs are errantly considered a retroverted acetabulum. To a more accurate and practical interpretation, in the native position (0° tilt and rotation), none of the false-positive COS can be determined by measuring the defined distance. The accuracy and diagnostic value of the distance between the coccyx and the pubic symphysis improved in proportion to the rise in the degree of tilt, so that by 10° of pelvic tilt, all of the false-positive COSs are discernible. The presence of the COS in aligned and properly positioned pelvic radiographs is attributed to the location of the anterior inferior iliac spine (AIIS) and its variable size and morphology, which can help the orthopaedic surgeon to evaluate the acetabular version. Other reports about the positive COS incorrectly aligned and positioned pelvis also confirmed that the sole use of the COS could be used as the rough estimate of acetabular retroversion [16, 21]. In brief, the COS can falsely appear as a normal hip variation even at a normal distance of the coccyx to the pubic symphysis and entire proper alignment and position. Of course, it does not express the retroverted acetabulum alone.

This study evaluated the COS in different positions using the pelvic models, which three-dimensional printers have made. In this manner, dynamic assessment and tactile manipulation through 3D models may better present a person's anatomy than what can be achieved with static image analysis. Likewise, it helps make the intended position more easily and more accurate. Furthermore, three-dimensional models provide a more intuitive understanding and detailed observation of skeletal anatomy. Prior reports investigated the diagnostic value of the COS in the cadavers' pelvis or had included prior imaging of the subjects who either had unilateral hip dysplasia or unilateral acetabular fractures and who had radiographically normal contralateral hips. It is worth mentioning that the use of cadavers increases the risk of malrotation [10, 21]. In pretaken photos, it is possible that the images are not taken precisely at the specified angle and position. Likewise, the impact of rotational alterations of the lumbar lordosis on pelvic tilt could not be corrected using prior radiographs. Furthermore, 3D models can potentially help preoperative decisions regarding the provision of more details of skeletal anatomy. It was reported that 3D models significantly helped to alter preoperative planning for osteoplasty in the femur acetabular impingement surgery [22]. Moreover, some investigations need frequent imaging, which is not ethical to perform using human models (such as excessive exposure to radiation). Likewise, we can eliminate the soft tissue and human model-dependent effect by using the 3D model. Therefore, more use of 3D models in the future may allow orthopaedic assessments to predict various complications of surgery and review new surgical methods without worrying about ethical problems.

The current study was not without limitations. Our sample size was small, so the findings cannot be generalized to the entire population and may lead to biased cases. It was a pilot study aimed at the bold potential capacity of 3D model printing in orthopaedics. Further studies with larger populations need to be performed to assess the clinical and statistical validities of 3D model reconstruction in the evaluation of conventional skeletal-related markers. The second limitation was ignoring the effect and contributions of the cartilage, capsule, labrum, and periarticular soft tissue structures. We only assessed the bony structures, while other structures such as muscles affect the position of the pelvis.

## 5. Conclusion

In conclusion, the presence of pelvic tilt during radiography can falsely demonstrate crossover signs, by pelvic tilt. Considering the coccyx's distance to the pubic symphysis for determining the standard pelvic radiographs, the COS is not accurate and valid enough to denote the acetabular retroversion from normal variation. Therefore, to diagnose acetabular retroversion, the COS must be accompanied by other radiologic markers, and in case of doubt, a pelvic CT scan is recommended for the definitive diagnosis of acetabular retroversion.

## Figures and Tables

**Table 1:** Effect of pelvic tilt on the presence of the crossover sign and distance between the tip of the coccyx and the symphysis pubis in pelvic radiography.

Pelvic no.	Sex	Age	Anteversion (degree)	0 degrees (true AP^b^)	Crossover sign/C-S distance^a^ (mm)				
					2 degrees	4 degrees	6 degrees	8 degrees	10 degrees
1	Male	34	12.3	−/10	−/13	+/19^*∗*^	+/22^*∗*^	+/27^*∗*^	+/32
2	Male	42	11.9	−/18	+/21^*∗*^	+/25^*∗*^	+/30^*∗*^	+/35	+/39
3	Male	50	12.8	+/24^*∗*^	+/30^*∗*^	+/34	+/39	+/44	+/47
4	Male	30	13.1	−/35	+/40	+/45	+/48	+/54	+/57
5	Female	25	9.7	−/28	−/33	−/36	+/40	+/46	+/48
6	Female	34	12.2	−/19	+/23^*∗*^	+/28^*∗*^	+/33	+/37	+/42
7	Female	25	11.5	+/20^*∗*^	+/24^*∗*^	+/29^*∗*^	+/33	+/38	+/42
8	Female	39	10.7	−/12	−/18	−/23	+/28^*∗*^	+/34	+/37
Mean (±S. D)			11.7 ± 1.1	20.7 ± 8.2	25.2 ± 8.7	29.9 ± 8.2	34.1 ± 8.1	39.4 ± 8.3	43 ± 7.7

^a^C-S distance, coccygeal tip-symphysis pubis distance; ^b^AP, anteroposterior. ^*∗*^Indicative of a positive crossover sign along with a normal coccygeal tip to symphysis pubis distance (10–30 mm). +, indicative of a positive crossover sign; −, indicative of a negative crossover sign. S. D., standard deviation.

**Table 2:** Sensitivity and specificity of plain radiographs in normal C-S distance at different angles of tilt in the diagnosis of retroversion according to the crossover sign.

Angle of tilt	Sensitivity (%)	Specificity (%)
0 degrees	100.0	80.0
2 degrees	80.0	66.6
4 degrees	60.0	66.6
6 degrees	42.8	72.7
8 degrees	12.5	88.9
10 degrees	0.0	100.0

## Data Availability

The data used to support the findings of this study are available from the corresponding author upon request.
